# Alterations in Cellular Energy Metabolism Associated with the Antiproliferative Effects of the ATM Inhibitor KU-55933 and with Metformin

**DOI:** 10.1371/journal.pone.0049513

**Published:** 2012-11-21

**Authors:** Mahvash Zakikhani, Miguel Bazile, Sina Hashemi, Shiva Javeshghani, Daina Avizonis, Julie St Pierre, Michael N. Pollak

**Affiliations:** 1 Department of Oncology, McGill University, Montreal, Quebec, Canada; 2 Division of Cancer Prevention, McGill University, Montreal, Quebec, Canada; 3 Goodman Cancer Research Centre, McGill University, Montreal, Quebec, Canada; 4 Division of Experimental Medicine, McGill University, Montreal, Quebec, Canada; 5 Department of Biochemistry, McGill University, Montreal, Quebec, Canada; Northwestern University, United States of America

## Abstract

KU-55933 is a specific inhibitor of the kinase activity of the protein encoded by *Ataxia telangiectasia mutated* (ATM), an important tumor suppressor gene with key roles in DNA repair. Unexpectedly for an inhibitor of a tumor suppressor gene, KU-55933 reduces proliferation. In view of prior preliminary evidence suggesting defective mitochondrial function in cells of patients with Ataxia Telangiectasia (AT), we examined energy metabolism of cells treated with KU-55933. The compound increased AMPK activation, glucose uptake and lactate production while reducing mitochondrial membrane potential and coupled respiration. The stimulation of glycolysis by KU-55933 did not fully compensate for the reduction in mitochondrial functions, leading to decreased cellular ATP levels and energy stress. These actions are similar to those previously described for the biguanide metformin, a partial inhibitor of respiratory complex I. Both compounds decreased mitochondrial coupled respiration and reduced cellular concentrations of fumarate, malate, citrate, and alpha-ketogluterate. Succinate levels were increased by KU-55933 levels and decreased by metformin, indicating that the effects of ATM inhibition and metformin are not identical. These observations suggest a role for ATM in mitochondrial function and show that both KU-55933 and metformin perturb the TCA cycle as well as oxidative phosphorylation.

## Introduction

DNA repair deficiency facilitates accumulation of mutations and accelerates carcinogenesis. These are features of the ataxia-telangiectasia syndrome, seen in patients with loss of function of ataxia telangiectasia mutated protein (ATM) [1], [2]. On the other hand, robust DNA repair capacity by cancer cells leads to resistance to therapies such as ionizing radiation that are intended to cause lethal DNA damage [3]. Small molecule ATM inhibitors [4] were developed in the context of the classic role of ATM in DNA repair, with the rationale that inhibition of DNA repair would increase efficacy of radiation therapy or cytotoxic drugs. The finding that inhibition of ATM by the small molecule kinase inhibitor KU-55933 has an antiproliferative effect [5] was unexpected in the context of the classic role of ATM as a tumor suppressor gene. However, there is recent evidence for novel functions of ATM [6], including participation in insulin signalling by an effect on protein translation regulator 4E-BP1 [7], regulation of response to oxidative stress [8]–[10], regulation of ribonucleotide reductase [11], and activation of the pentose phosphate pathway [12], [13]. Recent results [14], [15] provide evidence that KU- 55933 also inhibits the function of the organic cation transporter 1 (OCT1), which is known to be involved in cellular influx of several drugs, including metformin. In view of a prior report [16] that mitochondrial function is defective in fibroblasts from patients with ataxia-telangiectasia, we studied the effects of the small molecule inhibitor KU-55933 on cellular energy metabolism. We compared the effects of the ATM inhibitor to those of metformin, because this biguanide is known to be a growth inhibitor with a mitochondrial site of action, at respiratory complex I [17]–[21]. Other biguanides also inhibit mitochondrial function through incompletely described mechanisms [22].

## Results

### Effects of KU-55933 and/or Metformin on Cancer Cell Growth

**Figure 1:**
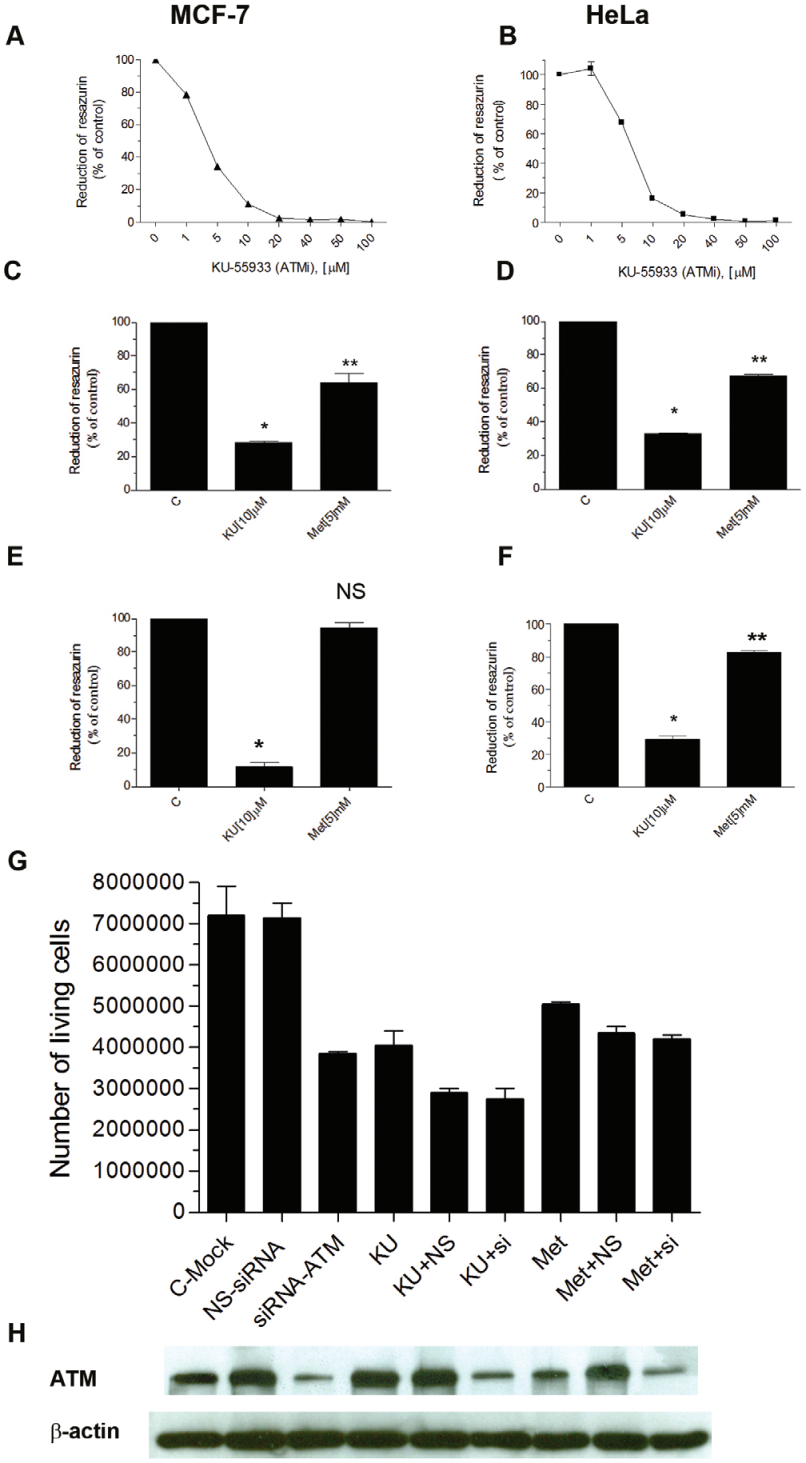
Growth inhibition by the ATM inhibitor KU-55933 and metformin. (**A**) MCF-7 (LKB^+/+^) and (**B**) HeLa (LKB^−/−^) cancer cells in exponential stages of growth were seeded into 96-well plates with 10% FBS and after 24 hrs exposed to increasing concentrations of KU-55933 (ATM inhibitor) in media containing 1% FBS for 72 hrs. Cell growth was estimated by Alamar Blue dye reduction (resazurin (3 µM)). Data are presented as mean ± S.E.M. from 3 independent experiments done in triplicate. (**C–F**) MCF-7 HepG2, HeLa and MCF-10A cells were growth inhibited by KU-55933 and metformin. Cells were seeded into 96-well plates in the presence of 1% FBS and after 24 hrs treated with KU-55933 (10 µM) or metformin (5 mM). Data are presented as mean ± S.E.M. from 4 independent experiments done in triplicate. * indicates a result significantly different from that obtained in the absence of KU-55933 or metformin as determined by 2-way ANOVA (*P*<0.0001). (**G**) MCF-7 cells were transfected with 50 nM ATM-siRNA or with control siRNA. Twenty-four hours after transfection, cells were treated with KU-55933 (10 µM) or metformin (5 mM) and incubated for 48 hrs in RPMI containing 1% FBS. Cell growth in each well was measured by counting cells using Trypan blue. Results using cell number or Alamar blue as endpoints yielded the same conclusions. Columns, mean of 3 independent experiments carried out in triplicate (n = 9); bars, S.E.M. (**H**) After transfecting MCF-7 cells with 50 nM ATM-siRNA or with control siRNA, cells were lysed and prepared for immunoblot analyses using antibodies against ATM. ß-actin is shown as a loading control.

Results of dose-response studies are shown in Figure 1A–B. Data shown in Figure 1C–F confirm that KU-55933 has antiproliferative effects on MCF-7, HepG2, HeLa and MCF-10A cell lines, as assessed by Alamar blue dye reduction. While this method is often used to estimate cell number, it actually is a measure of oxidative phosphorylation [16], so artefacts are possible if one is studying effects of an agent that influences cellular energy metabolism. Therefore, we confirmed an antiproliferative effect using cell number as an endpoint (Figure 1G). We also provide evidence in Figure 1G that an off-target effect of KU-55933 is unlikely, as an antiproliferative effect was also seen with ATM knockdown by siRNA. Western blot analysis confirmed reduced expression of ATM by siRNA but not by KU-55933 (Figure 1H). Our observation that the pharmacologic inhibition of ATM is growth inhibitory contrasts with prior reports [23], [24] that claimed ATM activation leads to apoptosis. However, these studies used non-specific pharmacologic strategies to activate ATM, so the induction of apoptosis cannot be definitely regarded as a consequence of ATM activation.

### Effects of KU-55933 and Metformin on Metabolism in MCF-7 Cells

**Figure 2:**
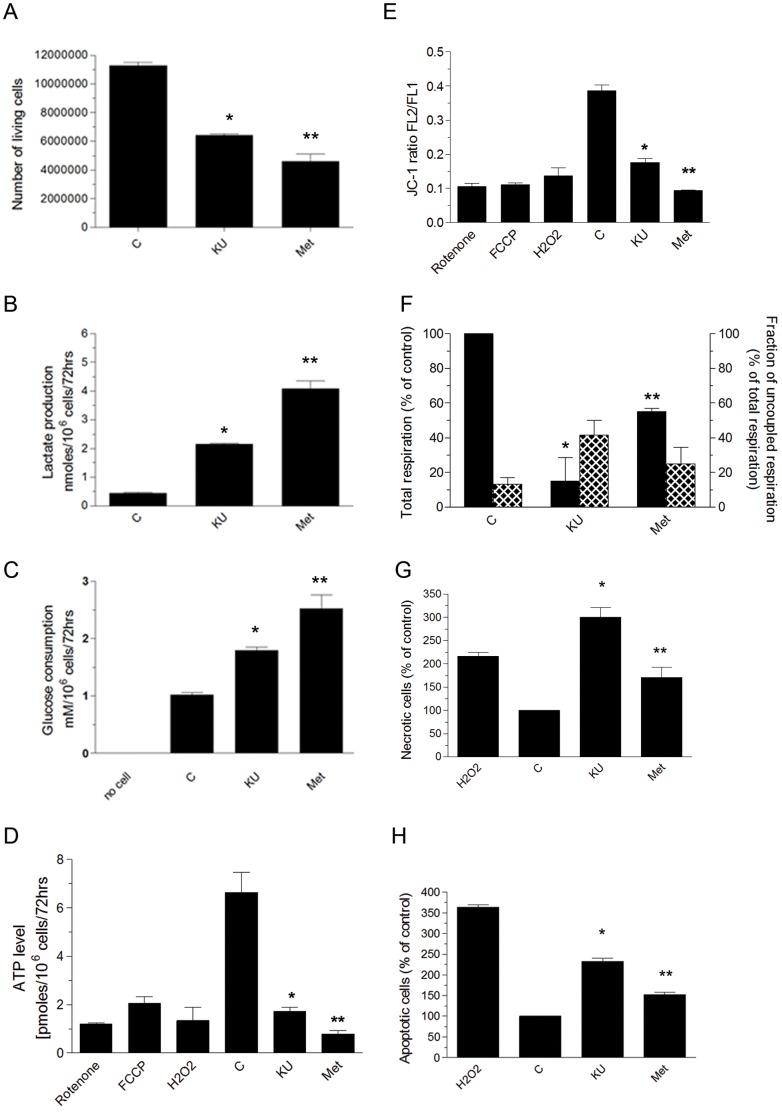
Effects of KU-55933 and metformin on metabolism in MCF-7 cells. Cells were exposed to KU (10 µM) or metformin (5 mM) for 72 hrs. (**A**) The effect of KU-55933 or metformin on viable cell number was measured by counting cells able to exclude Trypan blue. Cell number was significantly reduced by KU-55933 (*P = 0.0042) and by metformin (**P = 0.0011). (**B**) Lactate production was significantly increased in cells treated with KU-55933 (*P = 0.0218) or metformin (**P = 0.0012). (**C**) Glucose consumption was increased with exposure to either KU-55933 (*P = 0.0463) or metformin (**P = 0.0058) treated cells. (**D**) Both KU-55933 and metformin decreased ATP levels in MCF-7 cells. Results are the mean ± S.E (*n* = 4). (KU-55933 compared to control *P = 0.0015 and metformin compared to control ** *P* = 0.0005). (**E**) Cells were incubated with JC-1 (2 µM), or H_2_O_2_ (100 µM, used to activate ATM by oxidative stress), or rotenone (1 µM), or FCCP (1 µM). Mitochondrial membrane potential was probed with JC-1 and visualized by flow cytometry. Loss of mitochondrial membrane potential (ΔΨ) is indicated by a decrease in FL2/FL1 fluorescence intensity ratio **(see Figure S1 for flow cytometry data set)**. Results are expressed as mean ± S.E.M. (*n* = 4). KU-55933 (*P = 0.0003) and metformin (** *P*<0.0001) both significantly decreased ΔΨ. (**F**) Total cellular respiration (black bars, left y-axis) of MCF-7 cells treated with KU-55933 or metformin was compared with untreated cells. Results are the mean ± S.E.M. (KU-55933 compared to control *P = 0.0045, and metformin compared to control ** *P* = 0.0496). Uncoupled respiration was determined in the presence of oligomycin. The percentage of uncoupled respiration was calculated as: (uncoupled respiration/total mitochondrial respiration), and is shown by hatched bars, right y-axis. (**G**) KU-55933 or metformin treatment increased cell death (**see Figure S2 for flow cytometry data set)**. Bars represent percentage of necrotic cells. Results are expressed as the mean ± S.E.M. (*n* = 3) in duplicate (KU-55933 compared to control *P = 0.0005, and metformin compared to control **P = 0.0299). (**H**) KU-55933 or metformin treatment resulted in increased apoptosis **(see Figure S2 for flow cytometry data set).** Bars represent percentage of apoptotic cells. Results are expressed as the mean ± S.E.M. (n = 3) in duplicate (KU-55933 compared to control *P<0.0001, and metformin compared to control **P = 0.0458).

Figure 2A–C shows effects of KU-55933 and metformin on cell number, lactate production, and glucose consumption for MCF-7 cells. As expected, metformin decreased cell number, increased glucose consumption, and increased lactate production. These findings are consistent with previously reported actions of metformin as a growth inhibitor [17] with a mechanism related to partial inhibition of oxidative phosphorylation by an incompletely characterized action at respiratory complex I [19]–[21]. We observed that KU-55933 has previously unrecognized effects on each of these measurements similar to those of metformin, and we also observed that effects of metformin and KU-55933 together were additive for each of these endpoints.

Furthermore, as shown in Figure 2 (D–F), KU-55933 and metformin reduced ATP levels, mitochondrial membrane potential, and oxygen consumption, indicating inhibition of oxidative phosphorylation. Sequellae of exposure to either KU-55933 or metformin included both increased necrosis, as assessed by propidium iodide (PI) and increased apoptosis, as assessed by annexinV–FITC (Figures 2G and 2H). As recently reviewed [25], metformin can under certain conditions inhibit proliferation and enhance survival in an AMPK-dependent manner, but it can induce cell death if it is used in contexts where it causes severe ATP reduction.

Measurement of the percentage of cellular respiration uncoupled from ATP production (uncoupled respiration) (Figure 2F) revealed that metformin, apart from its previously partially characterized action on respiratory complex I, also increases the fraction of mitochondrial respiration devoted to uncoupled respiration, an action which would be expected to contribute to the decrease in ATP production caused by exposure to this agent. Unexpectedly, KU-55933 also increased the percentage of uncoupled respiration. Most importantly, our data demonstrate a significant inhibition in total cellular respiration devoted to ATP production by both metformin and KU-55933.

### Inhibition of ATM by KU-55933 Decreases SCO2 Levels in MCF-7 Cells

**Figure 3:**
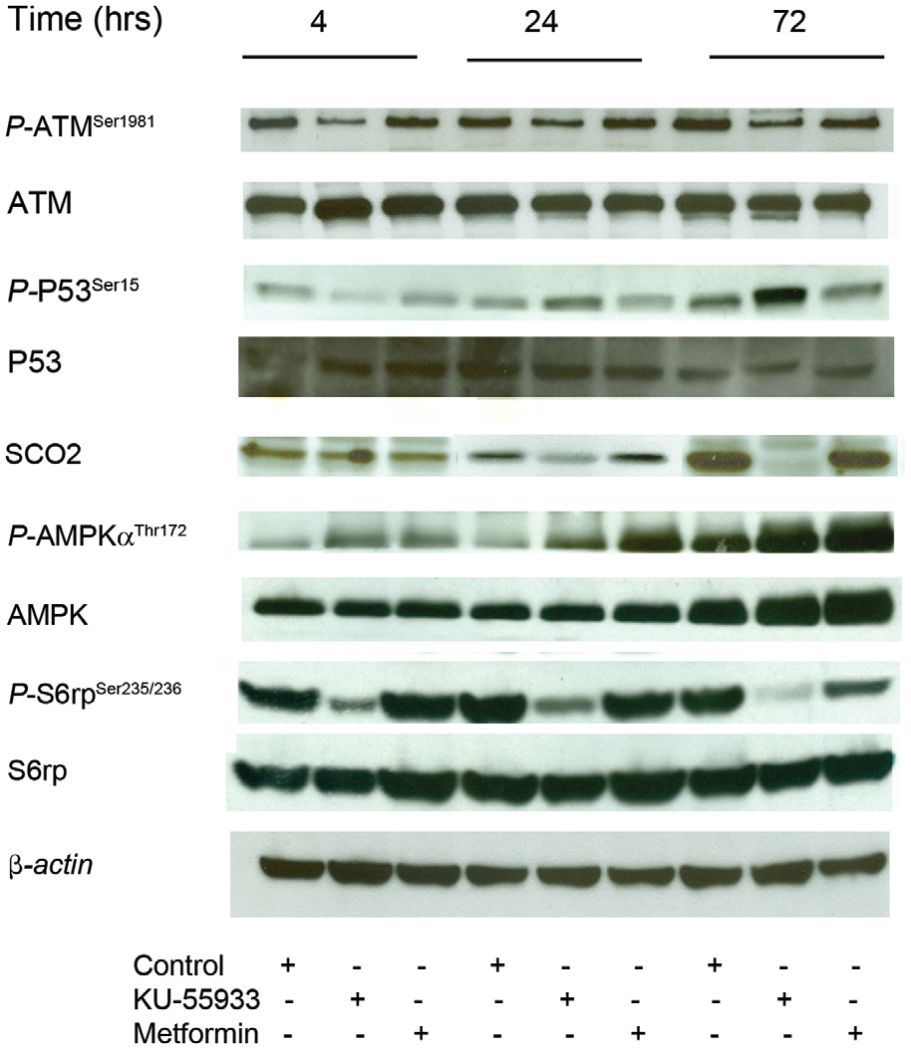
Inhibition of ATM by KU-55933 decreases SCO2 expression in MCF-7 cells. MCF-7 cells were exposed to KU-55933 (10 µM) for the indicated time. After harvesting, cells were lysed and prepared for immunoblot analyses using antibodies against SCO2, phospho-ATM (Ser^1981^), ATM, phosphorylated p53 (Ser^15^), p53, phospho-S6 (Ser^235/236^), S6rp, phospho-AMPK (Thr^172^) and AMPK. ß-actin is shown as a loading control. The results are representative of three individual experiments.

As ATM activates p53 [26] and p53 upregulates oxidative phosphorylation by increasing SCO2 [27], we considered the possibility that ATM inhibition may act to decrease p53 activation and therefore decrease SCO2 levels, which would be expected to decrease oxidative phosphorylation, as observed. This potential mechanism was appealing in view of a recent report [28] showing that in muscle, ATM inhibition reduces cytochrome c oxidase activity (by an unspecified mechanism), an action that is the expected consequence of SCO2 reduction, and which would result in the reduced mitochondrial function. As shown in Figure 3, KU-55933 had a major time-dependent effect in reducing SCO2 level in MCF-7 cells, consistent with this hypothesis. Figure 3 also demonstrates the expected effects of KU-55933 as an activator of AMPK secondary to energy stress, accompanied by a decline in S6 phosphorylation, in keeping with the previously described inhibitory effects of AMPK on mTOR by metformin [17].

### Effects of KU-55933 and Metformin on Metabolism and SCO2 Levels

**Figure 4:**
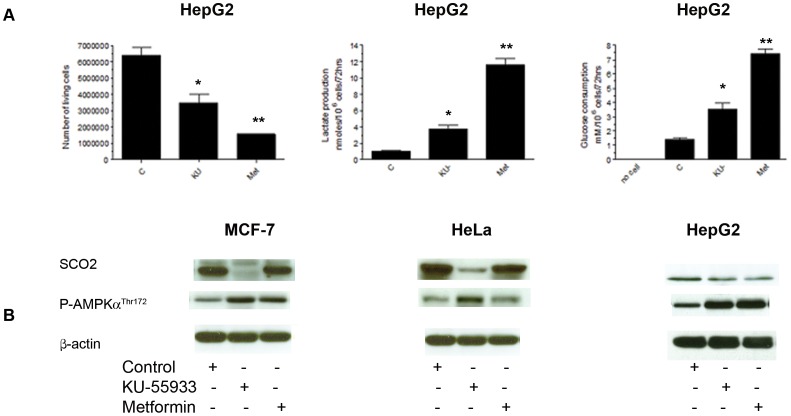
Effects of KU-55933 and metformin on cell number, lactate production, glucose consumption and SCO2 levels in different cancer cell lines. Cells were exposed to KU (10 µM) or metformin (5 mM) for 72 hrs. (**A**) The effect of KU-55933 or metformin on cell number was measured by counting cells able to exclude Trypan blue. Cell number was significantly reduced by KU-55933 (*P = 0.0394) and by metformin (**P = 0.0058). KU-55933 and metformin stimulated lactate production. Lactate production was significantly increased in cells treated with KU-55933 (*P = 0.0012) or metformin (**P = 0.0222). Glucose consumption was increased with exposure to either KU-55933 (*P = 0.0034) or metformin (**P = 0.0385) treated cells. (**B**) MCF-7, HeLa and HepG2 cells were exposed to KU-55933 (10 µM) or metformin (5 mM) for the indicated time. After harvesting, cells were lysed and prepared for immunoblot analyses using antibodies against SCO2, phospho-AMPK (Thr^172^). ß-actin is shown as a loading control. The results are representative of three individual experiments.

Figure 4A shows effects of KU-55933 and metformin on cell number, lactate production, and glucose consumption in HepG2 cells. Similar to the effects observed in the MCF-7 cell line, we also see an increase in glucose consumption, an increase in lactate production, as well as a decrease in cell number in the HepG2 cell line. Further studies did not support the view that this mechanism is universal. As shown in Figure 4B the KU-55933-induced decline in SCO2 levels was cell line specific, and HepG2 cells provide an example of growth inhibition, increasing glucose consumption, and lactate production induced by the ATM inhibitor, in the absence of a significant change in SCO2 level. We also found that in response to treatment with KU-55933, the LKB1-deficient cancer cell line, HeLa, exhibited AMPK-α phosphorylation. This indicates the existence of an LKB1-independent AMPK phosphorylation pathway.

### Effects of KU-55933 on HCT116 p53^+/+^ and HCT116 p53^−/−^ Cells

**Figure 5:**
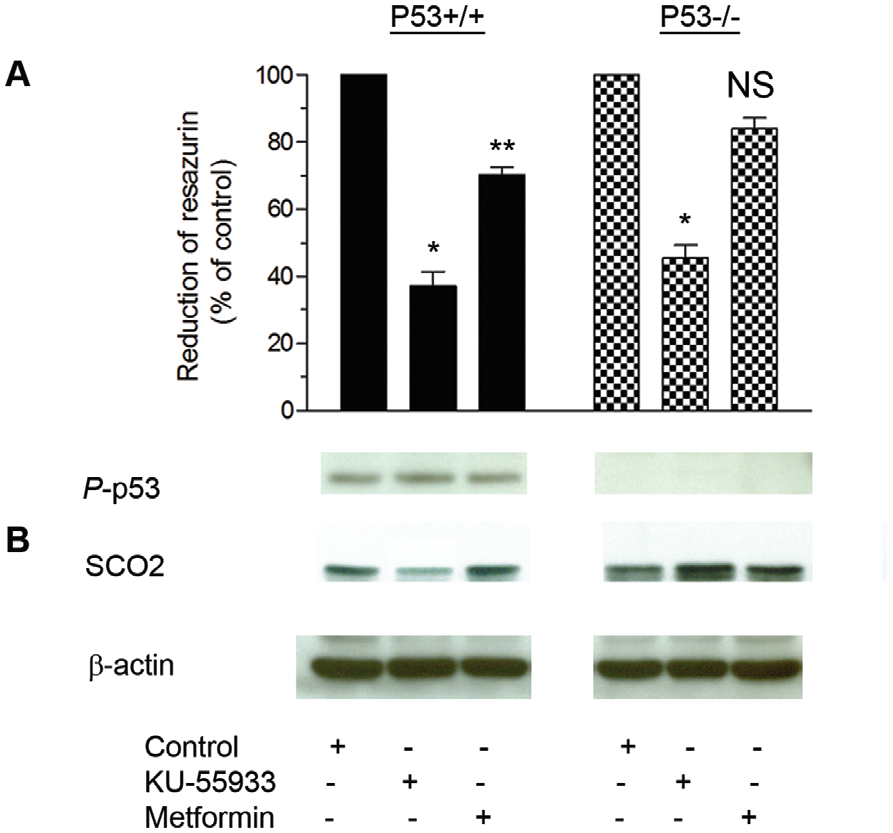
Effects of KU-55933 on HCT116 p53^+/+^ and HCT116 p53^−/−^ cells. Cells were seeded into 96-well plates with 10% FBS and after 24 hrs. exposed to KU-55933 (10 µM) or metformin (5 mM) in DMEM containing 1% FBS for 72 hrs. (**A**) Cell growth was estimated by Alamar Blue dye reduction. Results are presented as mean ± S.E.M. from 3 independent experiments in triplicate. HCT116 p53^+/+^ cell growth was significantly inhibited by both KU-55933 (*P<0.0001) and metformin (** *P* = 0.0013). For HCT116 p53^−/−^ cells, KU-55933 significantly inhibited growth (*P = 0.0002), but this effect was not seen with metformin exposure (*P* = 0.223). (**B**) Under the above conditions, after harvesting, cells were lysed and prepared for immunoblot analyses using antibodies against phosphorylated p53 (Ser^15^), and SCO2. ß-actin is shown as a loading control.

We examined the effects of KU-55933 on mitochondrial function as assessed by reduction of resazurin in isogenic p53 wild type and p53 loss of function HCT116 cells, and observed that even in the absence of p53, the kinase inhibitor reduced mitochondrial function, demonstrating that inhibition of ATM-dependent p53 activation with subsequent p53-mediated dependent SCO2 activation cannot account for the effect of KU-55933 on mitochondrial function (Figure 5). The ability of KU-55933 to inhibit oxidative phosphorylation in p53-null cells also argues against a mediating role of TIGAR, a p53-dependent mitochondrial regulator [29].

### Effects of KU-55933 and Metformin on TCA Metabolites

**Figure 6:**
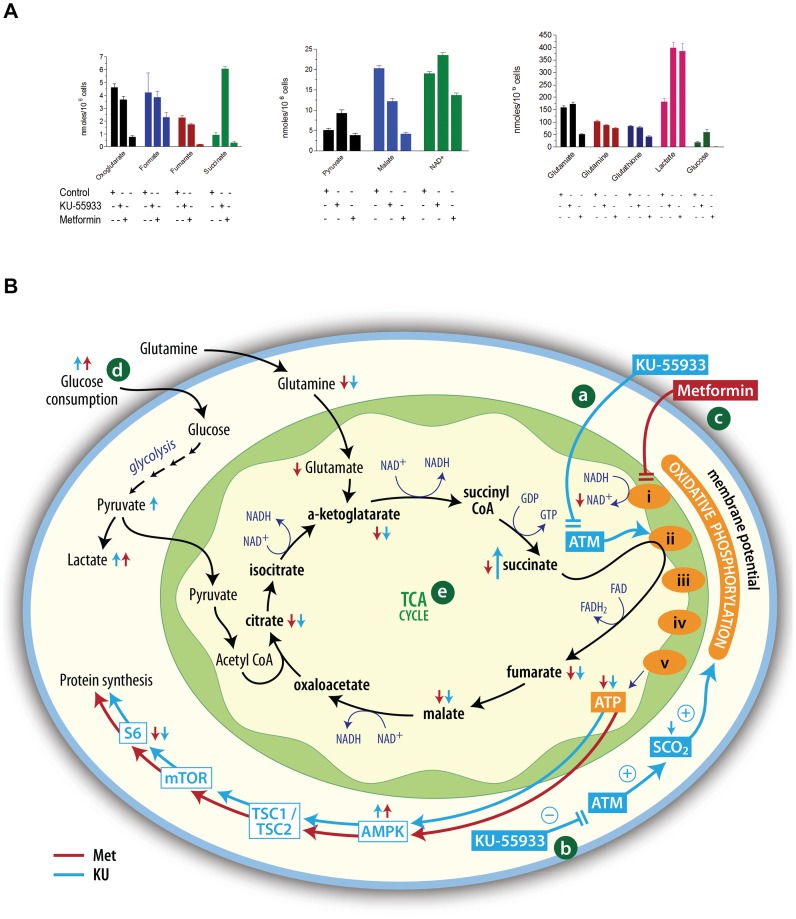
Effects of KU-55933 and metformin on TCA metabolites. (**A**) TCA metabolites were measured by NMR. *P* values for changes in TCA metabolites are shown in Table S1 (**B**) Interpretation of metabolic changes observed. *[a]* ATM is hypothesized to have a role in oxidative phosphorylation, effecting respiratory complex II. Therefore, the ATM inhibitor KU-55933 leads not only to reduced ATP production, but also to accumulation of succinate. *[b]* KU-55933 also may reduce oxidative phosphorylation by a mechanism involving SCO2, as discussed in the text. *[c]* Metformin acts to inhibit oxidative phosphorylation, but prior evidence together with our findings of decreased NAD^+^ suggest a site of action involving respiratory complex I. *[d]* Both KU-55933 and metformin exposure lead to increased glucose uptake and lactate production, consistent with a compensatory increase in glycolysis following decreased oxidative phosphorylation. *[e]* Our observations provide evidence for reduced concentrations of TCA cycle intermediates with exposure to either KU-55933 or metformin, but we postulate different reasons for this: metformin may reduce TCA cycle activity because of a reduction in supply of complex I-generated NAD^+^, while KU-55933 may act to inhibit conversion of succinate to fumarate. ATM, Ataxia Telangiectasia Mutated protein; SCO2, Synthesis of Cytochrome C Oxidase 2; AMPK, AMP-activated protein kinase; TSC1/TSC2, Tuberous Sclerosis 1/Tuberous Sclerosis 2; mTOR, Mammalian Target of Rapamycin complex 1; rpS6, ribosomal protein S6.

In order to better understand the consequences of KU-55933 and metformin on cellular energy metabolism, we measured intracellular levels of the metabolites indicated in Figure 6A and Table S1. Interestingly, both compounds increased intracellular lactate and glucose, consistent with data in Figure 2 concerning glucose absorption and lactate excretion. Both KU-55933 and metformin significantly reduced the concentrations of the TCA cycle intermediates fumarate, malate, citrate, and alpha ketoglutarate. The compounds differed with their effects on succinate level, which was increased more than 5-fold by KU-55933, but reduced by metformin. NAD^+^ levels were significantly reduced only by metformin. The underlying mechanisms require further study, but these data suggest that in the case of metformin, effects on respiratory complex I are important, and that the compound reduces generation of NAD^+^ by complex I. Thus, metformin may not only reduce oxidative phosphorylation, but also inhibit the TCA cycle via its effect on redox status, given that the TCA enzymes isocitrate dehydrogenase and alpha ketoglutarate dehydrogenase require NAD^+^. Although the effects of KU-55933 lead to many derangements similar to those seen with metformin, the lack of a significant effect on NAD^+^ and the greater than 5-fold increase in succinate levels seen with KU-55933 exposure raise the possibility of an effect on respiratory complex II (Figure 6B). Complex II oxidizes succinate to fumarate and reduces ubiquinone to ubiquinol. The former reaction is part of the TCA cycle, while the latter forms part of the respiratory chain of oxidative phosphorylation. It is conceivable that KU-55933 may directly or indirectly cause complex II dysfunction in a manner that reduces oxidative phosphorylation as well as conversion of succinate to fumarate, leading to accumulation of succinate and inhibition of the Krebs cycle.

### Subcellular Localization of ATM in MCF-7 Cells

**Figure 7:**
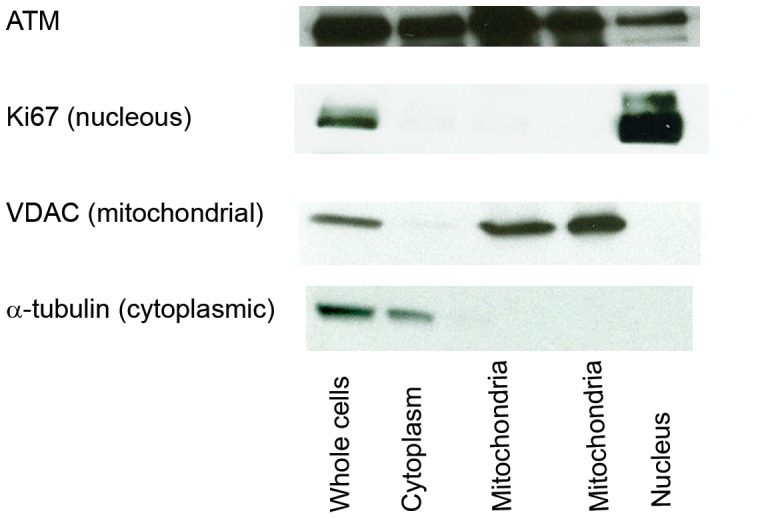
Subcellular localization of ATM. Total MCF-7 cell lysate and MCF-7 cells fractionated into cytoplasmic, nuclear and mitochondrial extracts were immunoblotted with ATM antibody, α-Tubulin (cytoplasmic marker), Ki67 (nuclear marker) and VDAC (mitochondrial marker). The results indicate ATM immunoreactivity in mitochondrial extracts that are negative for cytoplasmic and nuclear markers.

Our observations raise the possibility of a direct role for ATM in the mitochondria. While traditionally considered a nuclear protein, there is prior evidence [30] for cytoplasmic localization of ATM, but ATM has not previously been localized to mitochondria. We prepared a subcellular fraction highly enriched for mitochondria, and detected immunoreactivity to a mitochondrial marker VDAC and to ATM, but neither to the cytoplasmic marker tubulin nor the nuclear marker Ki67 (Figure 7).

## Discussion

ATM-related proteins are ancient in evolutionary terms [31], and our findings add to recent evidence suggesting that these kinases have important functions in addition to those initially described that are related to DNA repair [2]. A prior study of fibroblasts obtained from a patient with the ataxia telangiectasia syndrome [16] provided early evidence that ATM deficiency is associated with abnormalities in mitochondrial function that could not be accounted for by DNA repair deficits. Our studies extend this work by showing that pharmacologic inhibition of ATM with KU-55933 results in reduced mitochondrial membrane potential, reduced coupled respiration, and reduced ATP levels, while increasing glucose uptake and lactic acid production. These actions are similar to those of metformin, a compound known to partially inhibit respiratory complex I. We speculate that the increased glucose uptake and lactic acid production are a consequence of increased glycolysis that partially compensates for the decrease in mitochondrial ATP production in the setting of loss of function of ATM, suggesting that neoplasms involving loss of function of ATM will exhibit a “Warburg” metabolic phenotype.

Although inhibition of ATM by KU-55933 decreased expression of SCO2 (a protein required for cytochrome c oxidase assembly) in a p53-dependent fashion, the compound retained antiproliferative activity in p53-null cells, indicating that the actions of p53 are dispensable for the effects of KU-55933 on metabolism and proliferation. ATM has other substrates than p53 [32], [33], including Sp1 [34], that may alter nuclear gene expression patterns in ways that influence metabolism. Alternatively, the evidence that ATM is present in mitochondria raises the possibility that it may play a more proximal role in regulating oxidative phosphorylation. The significant increase in succinate concentration associated with exposure to the ATM inhibitor allows speculation that this drug may have a direct or indirect effect that compromises the activity of respiratory complex II (succinate:ubiquinone oxidoreductase) (Figure 6–2). Little is known about potential regulation of the activity of this complex by phosphorylation [35], but we did not detect a consensus sequence for the kinase activity of ATM against any of the subunits, arguing against a direct effect of ATM on this complex, despite the evidence for a requirement of ATM for optimum mitochondrial function.

In keeping with the fact that mitochondrial toxins are commonly found in nature, metformin is a respiratory complex I inhibitor derived from plant guanidines [36], and the plant toxin 3-nitropropionic acid [37] as well as the atpenin antibiotics [38] are complex II inhibitors. While complete inhibition of oxidative phosphorylation by agents such as cyanide is obviously lethal [39], and crude attempts to inhibit oxidative phosphorylation in cancer patients with cyanogenic molecules such as amygdalin derivatives are discredited [40], attention is being given to the possibility that reduction of oxidative phosphorylation by biguanides such as metformin may be useful in cancer treatment [25], [41].

Despite the fact that metformin has credentials as a complex I inhibitor, it is known to have a favorable safety profile in the treatment of type II diabetes [21], [25], [41]. It has both AMPK-dependent [17], [42] and AMPK-independent [43], [44] antiproliferative actions. The safety and efficacy of inhibitors of oxidative phosphorylation is critically dependent on their cellular and whole organism pharmacokinetic profiles, and their potential usefulness as antineoplastic agents will depend in part on uptake by neoplastic tissue [25]. It remains to be determined if the effects of KU-55933 and metformin we observed on levels of TCA cycle intermediates, uncoupled respiration, and oxidative phosphorylation are achievable *in vivo*.

The basis for the surprising observation that polymorphisms in the ATM locus influence efficacy of metformin in diabetes treatment [45] remains obscure. It has been pointed out (14) that laboratory evidence used to support the genetic results for this funding is open to question, as the ATM inhibitor used in the experiments may inhibit metformin influx into cells [46]. More importantly in the context of our results, however, is the fact that KU-55933 was previously noted to enhance the phosphorylation of AMPK, a finding which was unexplained by the authors (14) but is consistent with our results.

There are recent precedents for regulation of metabolism by oncogenes and tumor suppressor genes [47], [48]. Our results add to the evidence that ATM is a regulatory kinase with relevance to cellular energy metabolism. While the classic tumor suppressor properties of ATM are related to a requirement for the protein for normal DNA repair, our results provide evidence that the antiproliferative consequences of ATM inhibition arise as a consequence of the novel role for ATM in mitochondrial function.

Pharmacologic inhibition of ATM as a therapeutic strategy to enhance efficacy of DNA-damaging cancer treatments has been proposed [4], but may involve risks related to facilitation of carcinogenesis. Our studies do not address this issue, but rather make use of the ATM inhibitor KU-55933 to reveal previously unrecognized functions of ATM that relate to mitochondrial function rather than DNA repair.

## Materials and Methods

### Chemicals

Cell culture materials were obtained from Invitrogen (Burlington, ON, Canada). Anti-phospho AMPKα (Thr^172^), anti-AMPKα, anti-ATM, anti-phospho p53, anti-p53, anti-phospho S6 ribosomal protein (Ser^235/236^), anti-S6 ribosomal protein, anti-VDAC (voltage-dependent anion channel), anti-α-tubulin, and anti-ß-actin were purchased from Cell Signaling Technology (Beverly, MA), anti-phospho-ATM (Ser^1981^) and anti-SCO2 from Abcam (Cambridge, MA), and anti-Ki67 from Novus Biologicals (Oakville, ON, Canada). Horseradish peroxidase-conjugated anti-rabbit IgG, anti-mouse IgG, and enhanced chemiluminescene (ECL) reagents were from Pharmacia-Amersham (Baie d’Urfé, QC, Canada). Metformin (1, 1-Dimethylbiguanide hydrchloride), rotenone and FCCP, (Carbonyl cyanide 4-(trifluoromethoxy)phenylhydrazone) were purchased from Sigma-Aldrich (Oakville, ON, Canada), and KU-55933 from Calbiochem-EMD Biosciences, Inc (La Jolla, CA). siRNA against ATM and LKB1 and negative control siRNA (Alexa Fluor 488) were purchased from QIAGEN (Mississauga, ON, Canada), JC-1 (5,59,6,69-tetramethylbenzimidazolcarbocyanine iodide) from eBiosience (San Diego, CA).

### Cell Lines and Culture Conditions

MCF-7(breast), HeLa (cervical), HepG2 (hepatom) and MCF-10A (untransformed human breast epithelial) cell lines were purchased from American Tissue Culture Collection (ATCC) (Manassas, VA). HCT116 p53^+/+^ and HCT116 p53^−/−^ (colorectal) (generously provided by Dr. Russell Jones, McGill University and have been described previously in [49]) were cultured in RPMI 1640 or DMEM, supplemented with 10% fetal bovine serum (FBS) and 100 units/ml gentamycin at 37°C and 5% CO_2._ Cells were passaged by 0.25% Trypsin-EDTA when they reached ∼ 80% confluence.

### Cell Proliferation Assay

The effect of metformin or KU-55933 on cell lines was evaluated by the resazurin assay to measure overall mitochondrial respiration rates (Alamar Blue), (Biosource International, Camarilo, CA). Cells were plated at 3−5×10^3^ per well in triplicate in 96-well plates and incubated in medium containing 10% FBS. After 24 hrs, the complete medium was replaced with test medium containing vehicle control or various doses of metformin or KU-55933 for 72 hrs at 37°C. Alamar Blue was then added to plates which were incubated at 37°C according to the methods provided by the supplier and a colorimetric change measured the reduction of resazurin as an indicator of overall mitochondrial function which correlated with cell number.

### ATP Measurements

Cellular ATP levels were measured using the Invitrogen ATP Determination Kit A22066 (Invitrogen). Cells were treated in 1% FBS RPMI 1640 in the absence or presence of KU-55933 or metformin for 72 hrs. The kit was used as per the manufacturer’s instructions, with 3×10^5^ cells per well. Measurements were done in triplicate.

### Measurements of Glucose Consumption

Cells were cultured in complete medium with 10% FBS. After 24 hrs, the complete medium was replaced with test medium in the absence or presence of KU-55933 or metformin. Cells were incubated for 72 hrs and the culture medium was then collected and analyzed for measurement of glucose and lactate concentrations using colorimetric kits according to manufacturer’s instructions. Glucose levels were determined using a Glucose assay kit (Eton Bioscience, Inc**.**, Cambridge, MA). Glucose consumption was determined from the difference in glucose concentration compared to control. Results were normalized to cell-free media and to the number of cells.

### Lactate Production Assay

Lactate levels were determined in 10 µl culture medium collected from treated cells and results were standardised with the number of cells. Lactate was calculated using a Lactate Kit (BioVision, Inc., San Francisco, CA).

### Flow Cytometry for Apoptosis Induction and Cell Death Analysis

After 72 hrs treatment adherent cells were briefly trypsinized, detached, combined with floating cells from the original growth medium, centrifuged, and washed twice with Phosphate-Buffered Saline (PBS). Approximately 10^6^ cells (for each condition) were stained for 30 min with annexinV–FITC and propidium iodide (PI) using the AnnexinV–FITC kit (Invitrogen). Analysis was conducted on a FACSCalibur flow cytometer (BD Biosciences, Burlington, MA) with CellQuest software (BD Biosciences Immunocytometry Systems, Franklin Lakes, NJ). All apoptosis tests were conducted in triplicate and results shown are representative of 3 independent experiments.

### Mitochondrial Membrane Potential

To determine mitochondrial membrane potential JC-1 non-toxic fluorescence probe was dissolved in tissue culture grade dimethyl sulfoxide (DMSO) at a concentration of 1 mg/ml. After treatments, cells were probed with JC-1 and the mitochondrial membrane polarization changes were measured as described [50]. Cells treated with H_2_O_2_ (used as an ATM activator), rotenone (an inhibitor of complex I), or carbonyl cyanide m-chlorophenylhydrazone (FCCP) (an uncoupler of oxidative phosphorylation that abolishes the mitochondrial membrane proton gradient) were dissolved in DMSO and the solutions were added to culture medium to final concentrations as described in each experiment.

### Cell Transfection

MCF-7 cells were transfected with small interfering RNA (siRNA) targeting the ATM, LKB1, and AMPKα or a negative control siRNA using Pipette-type electroporator (MicroPorator MP-100, Digital Bio Technology Co., Ltd., Seoul, Korea) as described by the manufacturer’s instructions. Cells cultures were incubated for 24 hrs with various concentrations of siRNA prior to KU-55933 or metformin treatment.

### Protein Extraction and Western Blot Analysis

Cells were washed three times with ice-cold phosphate-buffered saline (PBS) and lysed in 100–400 µl lysis buffer (20 mM Tris HCl (pH 7.5)), 150 mM NaCL, 2.5 mM sodium pyrophosphate, 1 mM ß-glycerol phosphate, 1 mM Na_3_VO_4,_ 1 mM EGTA, 1% Triton, and Complete Protease Inhibitor Cocktail Tablet from Roche Diagnostic (Laval, QC, Canada). Cell debris was removed by centrifugation at 14,000X rpm for 20 min at 4°C. Following the assay for total protein (Bio-Rad, Mississauga, ON, Canada), clarified protein lysates from each experimental condition (40–50 µg) were boiled for 5 min and subjected to electrophoresis in denaturing 8% SDS-polyacrylamide gel for ATM, 12% for SCO2, or 10% SDS-PAGE for other proteins. Separated proteins were transferred to a nitrocellulose membrane and after blocking, the membranes were probed with antibodies of interest. In some cases, developed blots were stripped in stripping buffer (62 mM Tris HCL (pH 6.8), 100 mM ß-mercaptoethanol, 2% SDS) to confirm equal protein loading. Horseradish peroxidase-conjugated anti-rabbit IgG and anti-mouse IgG were used as secondary antibodies. The position of proteins was visualized using the enhanced chemiluminescene reagent ECL.

### Mitochondrial Extraction

Mitochondrial isolation was achieved by using the mitochondrial isolation protocol [51]. Mitochondrial pellets were lysed and protein concentration was determined with the Pierce® BCA Protein Assay Kit (Thermo Scientific), with bovine serum albumin (BSA) as a standard.

### Cellular Respiration Assay

Cells were rinsed, trypsinized, and spun twice at 1200 rpm for 5 min and resuspended in assay medium (PBS, sodium pyruvate (1 mM), glucose (25 mM), BSA 2% (w/v)). Cell viability was determined using trypan blue exclusions. Respiration in whole cells was measured using 1 million cells/ml (suspended in assay medium at 37°C), which were placed into the chamber of a Clark-type oxygen electrode (Rank Brothers, Cambridge, UK). Total respiration was assessed in the absence of inhibitors; while proton leak was measured using the ATP synthase inhibitor oligomycin (2.5 µg/1 × 10^6^ cells) and non-mitochondrial respiration using myxothiazol (12.2 µg/1 × 10^6^ cells).

### Cell Growth for NMR

MCF-7 Cells were plated at 1×10^6^ per *petri* in 10 replicates and incubated in medium containing 10% FBS. After 24 hrs, the complete medium was replaced with test medium containing vehicle control or metformin or KU-55933 at 37°C. Three MCF-7 plates were not extracted but used for cell counting and protein analysis. The average cell counts were 3.37 million per plate for the control cells, 1.9 million per plate for the KU-55933 treated cells and 2.1 million per plate for the metformin treated cells. These counts were used to normalize the NMR and GC/MS metabolite quantitation.

### NMR Sample Preparation

MCF-7 cells were extracted as described previously [52]. Briefly, tissue culture plates were removed from the incubator and 2 mL of the spent media was collected and placed in an eppendorf tube. The remaining media was aspirated off the plate to waste. The plated cells were washed three times with ice cold isotonic saline solution. Volumes of 500 µL 80% methanol (prechilled to −20°C) were added to the plates on ice. The cells were scraped off the plates and deposited in eppendorf tubes. The plates were rinsed with second 500 µL aliquots of cold 80% methanol and added to the cells. The cells were then lysed by 5 min of sonication, 30 sec on 30 sec off, on ice using Bioruptor UCD-200TM-EX Sonicator (Diagenode, Denville, NJ, USA). The homogenates were then spun down in a 4°C micro centrifuge for 10 min at 13,000 g and the supernatants were removed to new eppendorf tubes. The extracts were dried in a pre-cooled vacuum centrifuge (Labconco Corp. Kansas City, MO, USA) operating at −4°C and stored at −80°C until the day of NMR analysis.

For NMR analysis, cell extracts were re-suspended in 220 µL ^2^H_2_O containing 0.2 mM DSS (4,4-dimethyl-4-silapentane-1-sulfonic acid), the chemical shift and concentration standard 0.1 mM DFTMP (difluorotrimethylsilanylphosphonic acid) an internal pH standard [53] and 0.01 mM sodium azide. The pH of each sample was manually adjusted to an uncorrected pH of 6.8+/−0.1 with HCl or KOH as needed.

Medium samples were ultra-filtered using pre-rinsed 3 kDa cut off filters (Nanosep ultra filter, Pall Corp. Port Washington, NY USA). A volume of 195 µL was removed to a separate tube containing 22 µL of a 2 mM DSS and 1 mM DFTMP solution in ^2^H_2_O. The pH of each medium sample was manually adjusted to a pH of 6.8+/−0.1 as before. The samples were then transferred to 3 mm NMR tubes (Wilmad, Buena, NJ, USA) for analysis.

### NMR Data Acquisition & Analysis

NMR data collection was performed on a 500 MHz Inova NMR system (Agilent Technologies, Palo Alto, CA, USA) equipped with an HCN cryogenically cooled probe operating at 25 K. One-dimensional NMR spectra of samples were collected using the first increment of the standard NOESY experiment supplied with the instrument. All spectra were recorded at 25°C with a mixing time of 100 ms, 256 transients for cell extracts and 32 transients for media extracts were recorded with 8 equilibration pre-scans, a spectral window of 12 ppm centered on the residual water which was suppressed by a low power pre-saturation pulse during both the mixing time and 2 sec relaxation delay. The acquisition time was set for 3 sec for a total scan recycle time of 5 sec. The same pre-saturation strength and gain were used for all data acquisition (a slightly higher power pre-saturation pulse was used for all media samples) while the 90° pulse length was calibrated for each sample. Metabolite chemical shift assignments were confirmed by two-dimensional 75 ms mixing time total correlation spectroscopy (Z-filtered dipsi-Tocsy [54]) and by comparison to the Madison Metabolomics Consortium [55] and Human Metabolome data bases [56].

The one-dimensional data were processed with 128 k zero filling and exponential line broadening of 0.33 Hz before Fourier transformation. Targeted profiling of metabolites were achieved using a 500 MHz metabolite library from Chenomx NMR Suite 7.0 (Chenomx, Inc, Edmonton, AB, Canada), where area fit for the metabolite peaks were compared to that of the internal concentration standard (DSS) resulting in a concentration based on the Chenomx library compounds as described previously [57]. The amount of each reported metabolite was normalized to the number of cells per plate (nanomoles per million cells).

### Statistical Analysis

Prior to statistical analysis, data were square-root transformed to normalize the distribution and to obtain variance homogeneity. All experiments were performed at least in triplicate, and results are expressed as mean ± S.E.M. Statistical significance was evaluated using GLM Procedure, and least-squares means *post hoc* for multiple unpaired comparisons of means (LSMEANS statement with Bonferroni correction) was applied. All statistical analyses were performed using Statistical Analysis System software, version 9.2 (SAS Institute, Cary, NC). *P* values <0.05 were considered significant.

## Supporting Information

Figure S1 Loss of mitochondrial membrane potential (ΔΨ) as indicated by flow cytometry is seen by a decrease in FL2/FL1 fluorescence intensity ratio. Results indicate that treatment with KU-55933 or metformin decreases mitochondrial membrane potential of MCF-7 cells.
(TIFF)

Figure S2 Cytograms of PI uptake (ordinate) vs. annexin V binding (abscissa). Apoptotic (annexin V^+^/PI^−^), vital (V^−/^PI^−^), and damaged (annexin V^−/^PI^+^) cells are shown. Cells displayed an increase in cell death and apoptosis when treated with KU-55933 (*P<0.0001) or metformin (**P = 0.0155).
(TIFF)

Table S1 Significance of differences in metabolites levels in the MCF-7 cells treated with KU-55933 or metformin. (n = 9).
(DOCX)

## References

[1] Lavin MF (2008) Ataxia-telangiectasia: from a rare disorder to a paradigm for cell signalling and cancer. Nat Rev Mol Cell Biol 9: 759–769. 18813293 10.1038/nrm2514

[2] Savitsky K, Bar-Shira A, Gilad S, Rotman G, Ziv Y, et al (1995) A single ataxia telangiectasia gene with a product similar to PI-3 kinase. Science 268: 1749–1753. 7792600 10.1126/science.7792600

[3] Helleday T, Petermann E, Lundin C, Hodgson B, Sharma RA (2008) DNA repair pathways as targets for cancer therapy. Nat Rev Cancer 8: 193–204. 18256616 10.1038/nrc2342

[4] Hickson I, Zhao Y, Richardson CJ, Green SJ, Martin NM, et al (2004) Identification and characterization of a novel and specific inhibitor of the ataxia-telangiectasia mutated kinase ATM. Cancer Res 64: 9152–9159. 15604286 10.1158/0008-5472.CAN-04-2727

[5] Li Y, Yang DQ (2010) The ATM inhibitor KU-55933 suppresses cell proliferation and induces apoptosis by blocking Akt in cancer cells with overactivated Akt. Mol Cancer Ther 9: 113–125. 20053781 10.1158/1535-7163.MCT-08-1189

[6] Ditch S, Paull TT (2012) The ATM protein kinase and cellular redox signaling: beyond the DNA damage response. Trends Biochem Sci 37: 15–22. 22079189 10.1016/j.tibs.2011.10.002 PMC3259275

[7] Yang DQ, Kastan MB (2000) Participation of ATM in insulin signalling through phosphorylation of eIF-4E-binding protein 1. Nat Cell Biol 2: 893–898. 11146653 10.1038/35046542

[8] Shackelford RE, Innes CL, Sieber SO, Heinloth AN, Leadon SA, et al (2001) The Ataxia telangiectasia gene product is required for oxidative stress-induced G1 and G2 checkpoint function in human fibroblasts. J Biol Chem 276: 21951–21959. 11290740 10.1074/jbc.M011303200

[9] Guo Z, Kozlov S, Lavin MF, Person MD, Paull TT (2010) ATM activation by oxidative stress. Science 330: 517–521. 20966255 10.1126/science.1192912

[10] Alexander A, Cai SL, Kim J, Nanez A, Sahin M, et al (2010) ATM signals to TSC2 in the cytoplasm to regulate mTORC1 in response to ROS. Proc Natl Acad Sci U S A 107: 4153–4158. 20160076 10.1073/pnas.0913860107 PMC2840158

[11] Eaton JS, Lin ZP, Sartorelli AC, Bonawitz ND, Shadel GS (2007) Ataxia-telangiectasia mutated kinase regulates ribonucleotide reductase and mitochondrial homeostasis. J Clin Invest 117: 2723–2734. 17786248 10.1172/JCI31604 PMC1952633

[12] Kruger A, Ralser M (2011) ATM is a redox sensor linking genome stability and carbon metabolism. Sci Signal 4: e17. 10.1126/scisignal.2001959 21467295

[13] Cosentino C, Grieco D, Costanzo V (2011) ATM activates the pentose phosphate pathway promoting anti-oxidant defence and DNA repair. EMBO J 30: 546–555. 21157431 10.1038/emboj.2010.330 PMC3034007

[14] Yee SW, Chen L, Giacomini KM (2012) The role of ATM in response to metformin treatment and activation of AMPK. Nat Genet 44: 359–360. 22456732 10.1038/ng.2236 PMC3359140

[15] Woods A, Leiper JM, Carling D (2012) The role of ATM in response to metformin treatment and activation of AMPK. Nat Genet 44: 360–361. 22456733 10.1038/ng.2235

[16] Ambrose M, Goldstine JV, Gatti RA (2007) Intrinsic mitochondrial dysfunction in ATM-deficient lymphoblastoid cells. Hum Mol Genet 16: 2154–2164. 17606465 10.1093/hmg/ddm166

[17] Zakikhani M, Dowling R, Fantus IG, Sonenberg N, Pollak M (2006) Metformin is an AMP kinase-dependent growth inhibitor for breast cancer cells. Cancer Res 66: 10269–10273. 17062558 10.1158/0008-5472.CAN-06-1500

[18] Zakikhani M, Dowling RJ, Sonenberg N, Pollak MN (2008) The effects of adiponectin and metformin on prostate and colon neoplasia involve activation of AMP-activated protein kinase. Cancer Prev Res (Phila Pa) 1: 369–375. 10.1158/1940-6207.CAPR-08-0081 19138981

[19] Owen MR, Doran E, Halestrap AP (2000) Evidence that metformin exerts its anti-diabetic effects through inhibition of complex 1 of the mitochondrial respiratory chain. Biochem J 348 Pt 3: 607–614. PMC1221104 10839993

[20] Schafer G (1969) Site-specific uncoupling and inhibition of oxidative phosphorylation by biguanides. II. Biochim Biophys Acta 172: 334–337. 4304727 10.1016/0005-2728(69)90077-2

[21] Viollet B, Guigas B, Sanz GN, Leclerc J, Foretz M, et al (2012) Cellular and molecular mechanisms of metformin: an overview. Clin Sci (Lond) 122: 253–270. 22117616 10.1042/CS20110386 PMC3398862

[22] Turner N, Li JY, Gosby A, To SW, Cheng Z, et al (2008) Berberine and its more biologically available derivative, dihydroberberine, inhibit mitochondrial respiratory complex I: a mechanism for the action of berberine to activate AMP-activated protein kinase and improve insulin action. Diabetes 57: 1414–1418. 18285556 10.2337/db07-1552

[23] Chiu YJ, Hour MJ, Lu CC, Chung JG, Kuo SC, et al (2011) Novel quinazoline HMJ-30 induces U-2 OS human osteogenic sarcoma cell apoptosis through induction of oxidative stress and up-regulation of ATM/p53 signaling pathway. J Orthop Res 29: 1448–1456. 21425328 10.1002/jor.21398

[24] Ma E-L, Zhao D-M, Li Y-C, Cao H, Zhao Q-Y, et al.. (2012) Activation of ATM-Chk2 by 16-dehydropregnenolone induces G1 phase arrest and apoptosis in HeLa cells. Journal of Asian Natural Products Research In press. 10.1080/10286020.2012.694874 22694166

[25] Pollak M (2012) Metformin in cancer prevention and treatment: the end of the beginning. Cancer Discov 2: 778–790. 22926251 10.1158/2159-8290.CD-12-0263

[26] Canman CE, Lim DS, Cimprich KA, Taya Y, Tamai K, et al (1998) Activation of the ATM kinase by ionizing radiation and phosphorylation of p53. Science 281: 1677–1679. 9733515 10.1126/science.281.5383.1677

[27] Matoba S, Kang JG, Patino WD, Wragg A, Boehm M, et al (2006) p53 regulates mitochondrial respiration. Science 312: 1650–1653. 16728594 10.1126/science.1126863

[28] Patel AY, McDonald TM, Spears LD, Ching JK, Fisher JS (2011) Ataxia telangiectasia mutated influences cytochrome c oxidase activity. Biochem Biophys Res Commun 405: 599–603. 21266166 10.1016/j.bbrc.2011.01.075 PMC3055168

[29] Bensaad K, Tsuruta A, Selak MA, Vidal MN, Nakano K, et al (2006) TIGAR, a p53-inducible regulator of glycolysis and apoptosis. Cell 126: 107–120. 16839880 10.1016/j.cell.2006.05.036

[30] Barlow C, Ribaut-Barassin C, Zwingman TA, Pope AJ, Brown KD, et al (2000) ATM is a cytoplasmic protein in mouse brain required to prevent lysosomal accumulation. Proc Natl Acad Sci U S A 97: 871–876. 10639172 10.1073/pnas.97.2.871 PMC15423

[31] Morrow DM, Tagle DA, Shiloh Y, Collins FS, Hieter P (1995) TEL1, an S. cerevisiae homolog of the human gene mutated in ataxia telangiectasia, is functionally related to the yeast checkpoint gene MEC1. Cell 82: 831–840. 7545545 10.1016/0092-8674(95)90480-8

[32] Linding R, Jensen LJ, Ostheimer GJ, van Vugt MA, Jorgensen C, et al (2007) Systematic discovery of in vivo phosphorylation networks. Cell 129: 1415–1426. 17570479 10.1016/j.cell.2007.05.052 PMC2692296

[33] Matsuoka S, Ballif BA, Smogorzewska A, McDonald ER III, Hurov KE, et al (2007) ATM and ATR substrate analysis reveals extensive protein networks responsive to DNA damage. Science 316: 1160–1166. 17525332 10.1126/science.1140321

[34] Olofsson BA, Kelly CM, Kim J, Hornsby SM, Azizkhan-Clifford J (2007) Phosphorylation of Sp1 in response to DNA damage by ataxia telangiectasia-mutated kinase. Mol Cancer Res 5: 1319–1330. 18171990 10.1158/1541-7786.MCR-07-0374

[35] Tomitsuka E, Kita K, Esumi H (2009) Regulation of succinate-ubiquinone reductase and fumarate reductase activities in human complex II by phosphorylation of its flavoprotein subunit. Proc Jpn Acad Ser B Phys Biol Sci 85: 258–265. 10.2183/pjab.85.258 PMC3561849 19644226

[36] Witters LA (2001) The blooming of the French lilac. J Clin Invest 108: 1105–1107. 11602616 10.1172/JCI14178 PMC209536

[37] Sun F, Huo X, Zhai Y, Wang A, Xu J, et al (2005) Crystal structure of mitochondrial respiratory membrane protein complex II. Cell 121: 1043–1057. 15989954 10.1016/j.cell.2005.05.025

[38] Miyadera H, Shiomi K, Ui H, Yamaguchi Y, Masuma R, et al (2003) Atpenins, potent and specific inhibitors of mitochondrial complex II (succinate-ubiquinone oxidoreductase). Proc Natl Acad Sci U S A 100: 473–477. 12515859 10.1073/pnas.0237315100 PMC141019

[39] Way JL (1984) Cyanide intoxication and its mechanism of antagonism. Annu Rev Pharmacol Toxicol 24: 451–481. 6428300 10.1146/annurev.pa.24.040184.002315

[40] Milazzo S, Ernst E, Lejeune S, Boehm K, Horneber M (2011) Laetrile treatment for cancer. Cochrane Database Syst Rev 11: CD005476. 10.1002/14651858.CD005476.pub3 22071824

[41] Pollak M (2010) Metformin and other biguanides in oncology: advancing the research agenda. Cancer Prev Res (Phila) 3: 1060–1065. 20810670 10.1158/1940-6207.CAPR-10-0175 PMC2954412

[42] Shaw RJ, Lamia KA, Vasquez D, Koo SH, Bardeesy N, et al (2005) The kinase LKB1 mediates glucose homeostasis in liver and therapeutic effects of metformin. Science 310: 1642–1646. 16308421 10.1126/science.1120781 PMC3074427

[43] Foretz M, Hebrard S, Leclerc J, Zarrinpashneh E, Soty M, et al (2010) Metformin inhibits hepatic gluconeogenesis in mice independently of the LKB1/AMPK pathway via a decrease in hepatic energy state. J Clin Invest 120: 2355–2369. 20577053 10.1172/JCI40671 PMC2898585

[44] Kalender A, Selvaraj A, Kim SY, Gulati P, Brule S, et al (2010) Metformin, independent of AMPK, inhibits mTORC1 in a rag GTPase-dependent manner. Cell Metab 11: 390–401. 20444419 10.1016/j.cmet.2010.03.014 PMC3081779

[45] Zhou K, Bellenguez C, Spencer CC, Bennett AJ, Coleman RL, et al (2011) Common variants near ATM are associated with glycemic response to metformin in type 2 diabetes. Nat Genet 43: 117–120. 21186350 10.1038/ng.735 PMC3030919

[46] Minematsu T, Giacomini KM (2011) Interactions of tyrosine kinase inhibitors with organic cation transporters and multidrug and toxic compound extrusion proteins. Mol Cancer Ther 10: 531–539. 21252289 10.1158/1535-7163.MCT-10-0731 PMC3063525

[47] Vander Heiden MG, Cantley LC, Thompson CB (2009) Understanding the Warburg effect: the metabolic requirements of cell proliferation. Science 324: 1029–1033. 19460998 10.1126/science.1160809 PMC2849637

[48] Lemarie A, Grimm S (2011) Mitochondrial respiratory chain complexes: apoptosis sensors mutated in cancer? Oncogene 30: 3985–4003. 21625217 10.1038/onc.2011.167

[49] Bunz F, Dutriaux A, Lengauer C, Waldman T, Zhou S, et al (1998) Requirement for p53 and p21 to sustain G2 arrest after DNA damage. Science 282: 1497–1501. 9822382 10.1126/science.282.5393.1497

[50] Troiano L, Ferraresi R, Lugli E, Nemes E, Roat E, et al (2007) Multiparametric analysis of cells with different mitochondrial membrane potential during apoptosis by polychromatic flow cytometry. Nat Protoc 2: 2719–2727. 18007607 10.1038/nprot.2007.405

[51] Frezza C, Cipolat S, Scorrano L (2007) Organelle isolation: functional mitochondria from mouse liver, muscle and cultured fibroblasts. Nat Protoc 2: 287–295. 17406588 10.1038/nprot.2006.478

[52] Xu Q, Vu H, Liu L, Wang TC, Schaefer WH (2011) Metabolic profiles show specific mitochondrial toxicities in vitro in myotube cells. J Biomol NMR 49: 207–219. 21359514 10.1007/s10858-011-9482-8

[53] Reily MD, Robosky LC, Manning ML, Butler A, Baker JD, et al (2006) DFTMP, an NMR reagent for assessing the near-neutral pH of biological samples. J Am Chem Soc 128: 12360–12361. 16984154 10.1021/ja063773h

[54] Shaka AJ, Lee CJ, Pines A (1988) Iterative schemes for bilinear operators; application to spin decoupling. Journal of Magnetic Resonance 77: 274–293.

[55] Cui Q, Lewis IA, Hegeman AD, Anderson ME, Li J, et al (2008) Metabolite identification via the Madison Metabolomics Consortium Database. Nat Biotechnol 26: 162–164. 18259166 10.1038/nbt0208-162

[56] Wishart DS, Knox C, Guo AC, Eisner R, Young N, et al (2009) HMDB: a knowledgebase for the human metabolome. Nucleic Acids Res 37: D603–D610. 18953024 10.1093/nar/gkn810 PMC2686599

[57] Weljie AM, Newton J, Mercier P, Carlson E, Slupsky CM (2006) Targeted profiling: quantitative analysis of 1H NMR metabolomics data. Anal Chem 78: 4430–4442. 16808451 10.1021/ac060209g

