# Supplementary figures and images for: Alterations in Cellular Energy Metabolism Associated with the Antiproliferative Effects of the ATM Inhibitor KU-55933 and with Metformin
Source: PLoS One. 2012 Nov 21;7(11):e49513. doi: 10.1371/journal.pone.0049513 (PMC3504012; doi:10.1371/journal.pone.0049513)

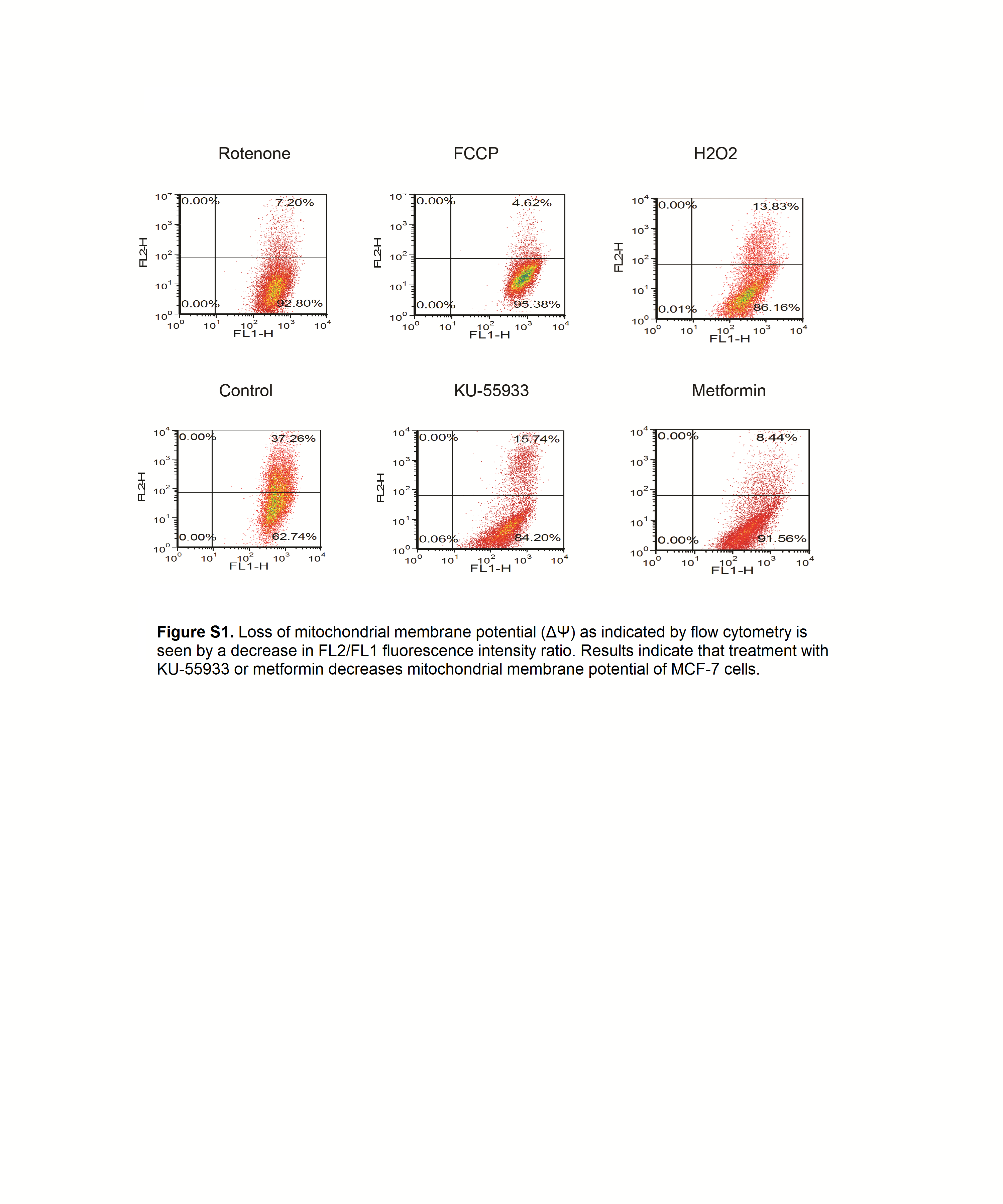

Supplement: Figure S1 — Loss of mitochondrial membrane potential (ΔΨ) as indicated by flow cytometry is seen by a decrease in FL2/FL1 fluorescence intensity ratio. Results indicate that treatment with KU-55933 or metformin decreases mitochondrial membrane potential of MCF-7 cells. (TIFF) [file pone.0049513.s001.tiff]

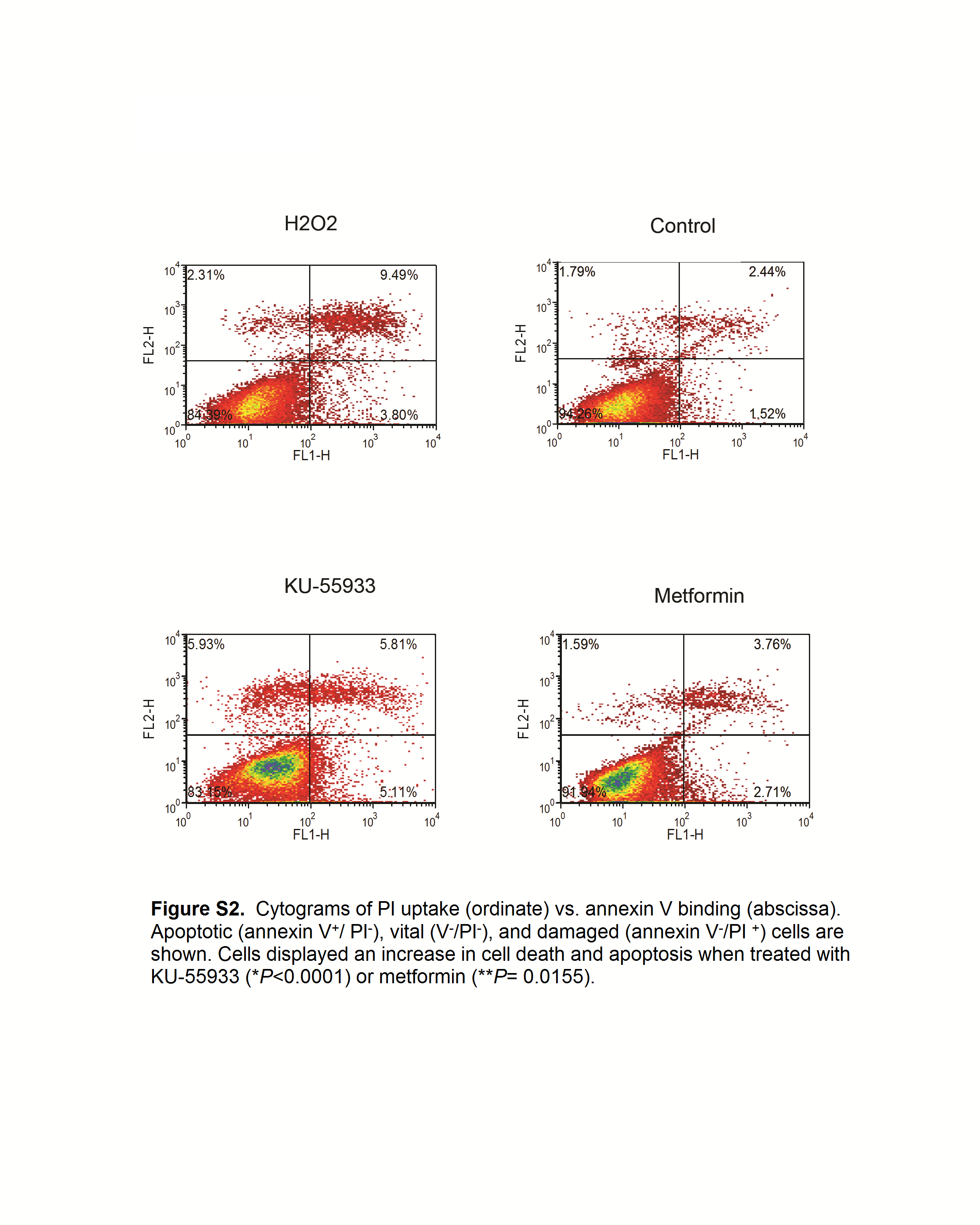

Supplement: Figure S2 — Cytograms of PI uptake (ordinate) vs. annexin V binding (abscissa). Apoptotic (annexin V+/PI−), vital (V−/PI−), and damaged (annexin V−/PI+) cells are shown. Cells displayed an increase in cell death and apoptosis when treated with KU-55933 (*P<0.0001) or metformin (**P = 0.0155). (TIFF) [file pone.0049513.s002.tiff]
