# Supplementary material for: Alterations in Cellular Energy Metabolism Associated with the Antiproliferative Effects of the ATM Inhibitor KU-55933 and with Metformin
Source: PLoS One. 2012 Nov 21;7(11):e49513. doi: 10.1371/journal.pone.0049513 (PMC3504012; doi:10.1371/journal.pone.0049513)
Supplement: Table S1 — Significance of differences in metabolites levels in the MCF-7 cells treated with KU-55933 or metformin. (n = 9). (DOCX) [file pone.0049513.s003.docx]

**Table S1**. Significance of differences in metabolites levels in the MCF-7 cells treated with KU-55933 or metformin. (n=9)

| **Metabolite**  **Treatment** | **Oxoglutarate** | **Formate** | **Fumarate** | **Succinate** |
| --- | --- | --- | --- | --- |
| **C *vs* KU** | ***P* = 0.0200** | **NS** | ***P* = 0.0078** | ***P* < 0.0001** |
| **C *vs* met** | ***P* < 0.0001** | **NS** | ***P* < 0.0001** | ***P* = 0.0275** |
| **KU *vs* met** | ***P* < 0.0001** | **NS** | ***P* < 0.0001** | ***P* < 0.0001** |

| **Metabolite**  **Treatment** | **Pyruvate** | **Malate** | **NAD+** |
| --- | --- | --- | --- |
| **C *vs* KU** | ***P* = 0.0002** | ***P* < 0.0001** | ***P* < 0.0001** |
| **C *vs* met** | ***P =*NS** | ***P* < 0.0001** | ***P* < 0.0001** |
| **KU *vs* met** | ***P* < 0.0001** | ***P* < 0.0001** | ***P* < 0.0001** |

| **Metabolite**  **Treatment** | **Glutamate** | **Glutamine** | **Glutathione** | **Lactate** | **Glucose** |
| --- | --- | --- | --- | --- | --- |
| **C *vs* KU** | ***P* = NS** | ***P* = 0.0105** | ***P* = NS** | ***P* < 0.0001** | ***P* = 0.0009** |
| **C *vs* met** | ***P* < 0.0001** | ***P* < 0.0001** | ***P* < 0.0001** | ***P* < 0.0001** | ***P* < 0.0001** |
| **KU *vs* met** | ***P* < 0.0001** | ***P =* NS** | ***P* < 0.0001** | ***P* = NS** | ***P* = NS** |
